# Learning to interpret one's own outcome as unjustified amplifies altruistic compensation: a training study

**DOI:** 10.3389/fpsyg.2013.00951

**Published:** 2013-12-19

**Authors:** Simona Maltese, Anna Baumert, Nadine Knab, Manfred Schmitt

**Affiliations:** ^1^Department of Psychology, University of Koblenz-LandauLandau, Germany; ^2^School of Psychology, University of Western AustraliaPerth, WA, Australia

**Keywords:** altruistic compensation, prosocial behavior, information processing, interpretational tendency, downward comparison

## Abstract

Interpretational tendencies in ambiguous situations were investigated as causal mechanisms of altruistic compensation. We used a training procedure to induce a tendency to interpret one's own advantages as unjustified. In a subsequent mixed-game, participants had to decide whether to invest their own money to compensate a victim of a norm violation. The amount of one's own resources invested as an altruistic compensation was enhanced after the training procedure compared to controls. These findings suggest that interpretational patterns with regard to injustice determine prosocial behavior. The training procedure offers a potential intervention strategy for enhancing altruistic compensation in bystander situations in which people must invest their own resources to restore justice.

## Introduction

Human behavior is guided by norms such as reciprocity and concerns for justice and fairness. People employ these standards to judge how right or wrong an action is (Kassin et al., 2010). Observed norm violations can trigger emotional reactions and motivate behavior to stop or redress the transgression. A particularly intriguing phenomenon appears in anonymous interactions in which an actor can expect no reward for a costly intervention. Investing one's own resources and taking risks to punish rule violations without direct self-interest is known as altruistic intervention. From an evolutionary standpoint, altruistic interventions serve important societal functions. Despite costs to the individual, the protection of norms maximizes the joint outcome of the group, thus raising its total fitness. To maintain this benefit, norm violation is socially sanctioned. Norm-protecting behavior can be seen as altruistic and in special cases prosocial because it guarantees the maintenance of a positive outcome for society, even at great cost and without direct benefit for the actor (e.g., Bowles and Gintis, 2004).

Previous research has revealed two types of altruistic interventions—altruistic punishment (e.g., Fetchenhauer and Huang, 2004) and altruistic compensation (e.g., Leliveld et al., 2012). In altruistic punishment, an uninvolved third party invests his/her own resources to stop or redress the perceived norm violation of a perpetrator. In altruistic compensation, a costly intervention is directed at abating the needs of victims of the norm violation (Lotz et al., 2011). Both are investigated by using the mixed-game (e.g., Fehr and Gächter, 2002). This experimental game involves three Persons A, B, and C. Person A gets 10 € with the opportunity to share any amount of this endowment with a powerless Person B. Person C has the costly option to invest any amount of his/her initial 10 € to compensate Person B by increasing his/her outcome and/or to punish Person A by reducing his/her outcome.

Results have shown that observers are willing to punish Person A (Fehr and Fischbacher, 2004; Nelissen and Zeelenberg, 2009) or to compensate the victim (Lotz et al., 2011; Leliveld et al., 2012) at their own expense when Person A makes an unequal offer to Person B (e.g., 10:0 €). However, people differ systematically in their proneness to altruistic interventions. Addressing the psychological processes driving altruistic punishment, research has suggested deterrence and just deserts as underlying motives (Carlsmith, 2006), whereas altruistic compensation requires a concern for the victim's outcome (Darley and Pittman, 2003). To date, the underlying mechanisms such as the interpretation of the situation and emotional reactions have not been investigated in detail.

It seems plausible that in ambiguous social situations, the interpretation as unjust or just leads to distinct and even opposite reactions. Accordingly, we aimed to uncover how interpretational processes in ambiguous situations contribute to the understanding of why some people invest their own resources to compensate a victim of another person's norm violation. Results will help to explain when and why altruistic compensation is displayed and potentially offer opportunities to enhance this behavior.

Evidence for the importance of interpretational processes for interpersonal behavior has been provided in research on negative social interactions. For aggression, theoretical models emphasize social information processing as a crucial factor. Thus, Crick and Dodge (1994) propose the “hostile attribution bias” as a psychological process that leads to aggressive behavior. It includes the tendency to interpret ambiguous situations as containing hostile intent toward oneself (Dodge and Crick, 1990; Tremblay and Belchevski, 2004). Research has shown that biased hostile interpretations in ambiguous situations increase the probability of aggressive behavior (e.g., Bushman and Anderson, 2002). Furthermore, in their meta-analysis, Orobio de Castro et al. (2002) reported a strong correlation between dispositional hostile attributions and behavioral aggression, indicating that people are generally more aggressive if they tend to interpret ambiguity as hostility.

Interpretational processes are stressed in a similar way by theoretical models of prosocial interpersonal behavior, including altruistic interventions. The seminal model by Latané and Darley (1970) proposes five stages of helping behavior as determined by psychological processes. Accordingly, an incident has to be witnessed and interpreted as an emergency. If an ambiguous situation is not interpreted as an emergency, it will not trigger helping behavior. Thus, interpretation is assumed to play a key role in determining subsequent behavioral reactions. However, there is only indirect evidence that interpretational processes are indeed crucial for prosocial behavior. First, in social psychology, research on the bystander effect has shown that an increasing number of inactive bystanders can reduce the probability that any single person will help. Assumedly, the reactions of others are used to interpret the ambiguous situation regarding the necessity of intervention (e.g., Latané and Darley, 1970; Fischer et al., 2006). Thus, perceiving the inaction of others can lead to an interpretation of the situation as less critical.

Second, there is indirect evidence for the relevance of interpretational processes for altruistic interventions in personality psychology. Specifically, stable and consistent individual differences in the perceptional readiness and emotional reactivity to injustice— namely, justice sensitivity (Schmitt, 1996; Schmitt et al., 2010)—have been identified as predictors of prosocial behavior (e.g., Gollwitzer et al., 2005; Baumert et al., 2013). Furthermore, justice sensitivity predicted altruistic punishment and altruistic compensation in the mixed-game (Lotz et al., 2011) described above. Importantly, it has been proposed that differences in justice sensitivity involve chronic interpretational tendencies that cause behavior. Preliminary empirical evidence showed that people high (compared to low) in justice sensitivity perceived an ambiguous situation as less just (Baumert and Schmitt, 2009) and were more ready to resolve ambiguous sentence fragments that yielded an unjust connotation (Baumert et al., 2012). In the mixed-game, when witnessing an unequal allocation by Person A, persons high in justice sensitivity may tend to interpret their own favorable outcome in the role of Person C as unjustified compared to Person B. Thus, their altruistic compensation may be directed at reducing the subjectively unjustified discrepancy between Person B and their own more favorable condition. The causal relevance of this interpretational pattern for triggering altruistic compensation has yet to be tested.

In sum, interpretational processes seem to play a crucial role in determining prosocial behavior, including altruistic interventions. As there is only indirect evidence, we aimed to fill this gap by experimentally testing interpretational processes as determinants of altruistic intervention.

## Importance of interpretational processes

When a bystander witnesses a perpetrator victimizing another person, social-comparison processes (Festinger, 1954) may be relevant for subsequent behavioral reactions. Observers can compare themselves with the perpetrator or the victim. Research suggests that altruistic punishment occurs if the observer compares his/her own standing with the perpetrator. Perceiving that the perpetrator has increased his/her status by breaking social norms can lead the observer to punish the perpetrator (Darley and Pittman, 2003; Fehr and Fischbacher, 2004). By contrast, little research has focused on the possibility that the observer of a norm violation interprets him- or herself as relatively privileged in comparison with a victim. In other words, the bystander may also engage in downward comparison. As we argue in the present paper, a focus on the victim might be a crucial determinant of altruistic compensation.

In general, downward comparison implies that one's focus is on a victim who is worse off. So far, downward comparisons have primarily been investigated from the perspective of a cognitive coping mechanism (e.g., Carmona et al., 2006) that helps to regulate negative emotions (see Buunk and Gibbons, 2007, for an overview) and maintain a positive self-perception (Taylor et al., 1990), especially in stressful situations (Buunk and Ybema, 1995). Regarding prosocial behavior, Yip and Kelly (2013) reported that downward comparison with targets whose performance was inferior to one's own led to reduced subsequent prosocial reactions and self-reported empathy toward others compared with controls who did not engage in social comparison. However, no research has systematically investigated whether the discrepancy between one's own and the other person's situation is interpreted as justified or not. Importantly, we assume that downward comparison can lead to an increase or a decline in prosocial behavior, depending on whether one's own relative advantage is perceived as legitimate. In this case, positive emotions that are not expected to lead to altruistic interventions should arise. By contrast, the interpretation of one's own advantage as illegitimate should result in the motivation to restore justice (Lockwood, 2002). Accordingly, when faced with an unequal allocation in the mixed-game, one could assume that altruistic compensation is motivated in the role of Person C, interpreting one's own outcome of 10 € as unjustified in comparison with Person B's outcome.

In sum, we argue that behavioral reactions in situations in which another person is victimized by a third person depend on the interpretation of one's own better conditions as unjustified or not. Until now, the causal role of the proposed interpretational pattern has not been formally tested. In the present study, we systematically manipulated interpretational tendencies in ambiguous social situations and tested their effect on altruistic compensation.

## Training of interpretational tendencies

In our study, we investigated whether the interpretation of ambiguous situations in terms of one's own unjustified advantages would cause altruistic compensation. In order to test the effect of interpretations on behavior, it is necessary to experimentally induce a specific interpretational readiness. Thus, as a first research question, we wanted to know whether it would be possible to induce short-term changes in interpretational tendencies. As a second step, we then tested the behavioral effects of these tendencies.

In research on anxiety, methods to manipulate interpretational biases with regard to threat have been successfully implemented (MacLeod and Cohen, 1993; Mathews and Mackintosh, 2000). Mathews and Mackintosh (2000) presented participants with sentences that remained ambiguous with regard to their valence until the last few words. The last few words resolved the ambiguity, indicating either a positive or a negative event. Importantly, the disambiguating words were presented as word fragments. Participants had to read the sentences and complete the fragments as quickly as possible. In order to induce a negative (vs. positive) interpretational tendency, participants repeatedly received ambiguous sentences that were always resolved as negative (or positive, respectively). Assumedly, participants will then adopt a readiness to interpret ambiguous sentences as negative (vs. positive), enabling them to complete the fragmented words more quickly. In order to assess the effectiveness of this training procedure, Mathews and Mackintosh employed a further set of ambiguous sentences: One half were resolved to be negative; the other half were resolved to be positive. Response latencies for fragment completion were measured. Results revealed that participants who were trained to interpret the ambiguous situations in a negative way were faster at completing the subsequent negative word fragments compared with participants trained to interpret the ambiguity in a positive way, and vice versa for positive word fragments.

In the present research, we modified this procedure by using sentences that were ambiguous with regard to justice or injustice. The sentences described situations in which the narrator receives a relative advantage, but whether this advantage is justified or not remains ambiguous. In the unjust training condition, the last words of the sentences resolved this ambiguity in the sense that the advantage was presented as unjustified and the narrator was seen as the beneficiary of an injustice. In the control condition, all sentences remained neutral with regard to in/justice. For all sentences, the final disambiguating words were presented as fragments. We instructed our participants to adopt the perspective of the narrator and to respond to the word fragments as quickly as possible.

In sum, we expected that repeatedly resolving ambiguous sentences to reflect unjust situations would induce a readiness to interpret one's own relative advantage as unjustified in ambiguous situations. This readiness was expected to appear in reaction times for the completion of word fragments in additional sets of ambiguous sentences. We expected that reaction times for completing disambiguating fragments that indicated an unjust benefit would be reduced in the unjust training condition compared with the control condition (Hypothesis 1).

If the training procedure was able to induce such an interpretational readiness, we further predicted effects on altruistic compensation in a subsequent mixed-game. When people were induced to interpret ambiguous situations in such a way that they readily saw their own positive outcome as unjustified, they were expected to be more prone to behave prosocially in a subsequent mixed-game in which they witnessed an unequal split of money between Person A and Person B. Accordingly, persons in the unjust training condition were expected to invest more of their own money to compensate Person B (Hypothesis 2a). In addition, we explored whether the training also affects altruistic punishment. Possibly, this is not the case because the tendency to interpret one's outcome as unjustified or not may not be a relevant process driving this kind of behavior. For altruistic punishment, a focus on the perpetrator might play an important role and not a focus on the victim that is worse off which is implied by the training procedure.

Furthermore, to rule out an alternative explanation for behavioral effects of the training, emotions were assessed. We expected the training not to affect emotions directly but to affect the interpretation of the situation.

## Method

### Sample

Undergraduate psychology students that had not joined a similar experiment were invited to participate in a study ostensibly on text comprehension. Seventy-eight persons (85% female) followed the invitation. Ages ranged from 18 to 46 years (*M* = 21.97, *SD* = 4.83). A posteriori power analyses revealed 1-β = 0.71 to detect medium size effect *d* = 0.5 in a two-tailed *t*-test. All participants spoke German fluently. In return for their participation, students received extra course credit.

### Procedure

When participants arrived at the laboratory session, they were seated at one of three separated workplaces and randomly assigned to one of two experimental conditions: the unjust training (*n* = 38) or the control condition (*n* = 39). Then participants worked on a word fragment completion task that contained training trials as explained below. Following the training trials, participants further completed unjust, just, and neutral probe fragments. These probes were designed to record how readily participants resolved an ambiguous sentence in a way that indicated an unjust or just outcome. The neutral probe fragments were designed to measure baseline reaction times to word fragments. Subsequently, participants played a mixed-game with three players as described earlier (Fehr and Gächter, 2002). Ostensibly at random, they were assigned to the role of Person C and witnessed an unequal split of money made by one of the players (10:0 €, see below). As a manipulation check, emotions were assessed. Participants then decided whether to invest their own money to compensate the person who had received nothing and/or to punish the person who had made the unequal split. Next, they were asked about their general beliefs regarding the mixed-game and the goal of the study. Finally, they were debriefed, thanked, and dismissed.

### Materials

All materials were presented in German. Here, we provide our own English translations.

#### Fragment completion task

Participants had to read the description of a student's day, imagining themselves in the narrator's situation. The description contained different passages that were presented sentence by sentence on the computer screen. The final words of some sentences were fragmented, and participants had to complete the missing letters (see below for examples). The instructions said to press a marked button to continue as quickly as possible once they had finished reading the sentence or knew the correct solution of a fragment. On the following screen, there was space to type in the missing letters. After completion, feedback was provided and either the word “correct” appeared in green letters or the word “wrong” in red letters. Participants first worked on three practice trials that were followed by 24 training trials intermixed with seven neutral filler items in order to mask the goal of the study.

***Training Fragments.*** Twenty-four passages were designed for the training and differed systematically between the experimental conditions.

In the *unjust training condition*, the first part of the passage was a description of a situation in which the individual is relatively advantaged, but it is ambiguous whether the advantage is justified or not. The fragmented final part of the passage indicated the relative advantage as not justified, presenting the narrator as the beneficiary of an injustice. In the *control condition*, passages described the same situations in a neutral way.

Examples are:

Unjust training condition: “I need to obtain an internship this semester. My father knows a famous marketing boss. Despite strong competition, I am given an internship in his company because the marketing boss tru_ts _y fa_her.” (correct response: “trusts my father”)

Control condition: “I need to obtain an internship this semester. My father suggests that I try the field of marketing. After extensive research, I find a company with an interesting position, and I read the j_b _rofile ca_efully.” (correct response: “job profile carefully”)

All materials were pretested. Each passage was rated by 30 raters (ages from 19 to 57, *M* = 24.57, *SD* = 8.22) on a scale with the anchors −3 (*very unjust*), 0 (*neither nor*), and 3 (*just*). Passages from the unjust training condition were given the lowest ratings (*M* = −1.60, *SD* = 0.61). The equivalent neutral passages were rated as rather neutral (*M* = 0.79, *SD* = 0.81). Ratings of the two sets of passages differed significantly from each other, *t*_(28)_ = −15.76, *p* < 0.01, *d* = −3.39.

#### Assessment of interpretational readiness

The training passages were followed by 12 probe fragments to assess interpretational readiness. All participants completed four just, four unjust, and four neutral probe fragments that were presented in a random order fixed across participants.

The structure of the probe passages was similar to the training trials. The narrator was presented as relatively advantaged, but whether the advantage was justified remained ambiguous. For the *just probes*, the fragmented words resolved the ambiguity in a way that presented the participant's own outcome as justified. For the *unjust probes*, the fragmented words resolved the ambiguity to indicate that the advantage was unjustified. The neutral probes had no justice-relevant content. Please note that the passages were not matched in length across probe types. Therefore, a comparison of reaction times across probe-types within groups is not informative because it is contaminated by different length of the probe sentences.

Examples are:

Just probe: “This evening at my gym, I would like to do a new workout that costs additional money. But in contrast to the other members, I do not have to pay becau_e I ha_e a vo_cher.” (correct response: “because I have a voucher”).

Unjust probe: “In the Department of Psychology, there is an open position for a research assistant, which is much sought-after. Although another fellow student applies, I get the position because I have connecti_ns to t_e depart_ent.” (correct response: “connections to the department”).

Neutral probe: “After the gym, I meet some colleagues at a cocktail bar. We talk about the first weeks of the semester and have lots of fun. Because we have not seen each other for a long time, we plan to spend so_e t_me tog_ther.” (correct response: “some time together”).

We recorded how quickly participants pressed the button after reading the fragmented sentence. In order to obtain a comparable reaction time measure for each participant, they were instructed to react as quickly as possible. After deciding what the correct solution was, they had to type the letters in on the next screen.

Probe passages were pretested together with the training passages (see above for sample characteristics). We selected four passages rated most unjust (*M* = −1.36, *SD* = 1.36) and four passages rated as neutral (*M* = 0.93, *SD* = 1.09), *t*_(26)_ = −8.13, *p* < 0.01, *d* = −0.36. Because the just probes did not differ significantly from the neutral probes, we modified those with ratings close to zero in order to make the just content more salient and pretested them again in a sample of 6 raters (ages from 24 to 28, *M* = 25.7, *SD* = 1.63). Even after modification, the four just probes differed only descriptively (*M* = 1.17, *SD* = 1.16) from the neutral probes, *t*_(32)_ = −0.36, *p* = 0.72, *d* = −0.17. Regarding the interrater agreement, there was a significant intra-class correlation between the ratings for just sentences, *r* = 0.21, *p* = 0.05. The raters did not significantly agree in their ratings for unjust, *r* = 0.07, *p* = 0.18, and neutral sentences, *r* = −0.03, *p* = 0.83.

#### Mixed-game

On the computer screen, participants were informed that they would be interacting with two other anonymous participants and that they would be randomly assigned to one of three roles, but all participants were actually given the role of Person C. They learned that initially, Person A would receive 10 € and would have the opportunity to share any amount of this money with an anonymous and powerless Person B. Person C was initially endowed with 10 € that could be invested to change the distribution made by Person A.

Participants were then told that they had the role of Person C. They were informed that Person A had allegedly decided to keep the entire 10 € for him/herself. Participants were informed that they could keep their own 10 € or use it to change the outcomes of Person A and/or Person B. Every investment of 1 € would lead to a 2 € change in the other persons' outcomes, meaning 2 € less for Person A or 2 € more for Person B. Participants were asked to decide about their possible investment regarding the punishment of Person A and the compensation of Person B. We assessed how much money participants invested in these two decisions.

***Manipulation Checks.*** Right before participants decided on whether to invest their money to punish and/or to compensate, their emotional reactions to the unequal allocation were assessed. Participants were asked to indicate on a response scale from 1 (*totally disagree*) to 6 (*totally agree*) how well each of 14 statements described their present feelings. Among filler items, we assessed outrage and guilt with four items each (outrage: e.g. “I am outraged about the distribution”; α = 0.91; guilt: “I feel guilty”; α = 0.73).

## Results

### Effects of training on interpretational readiness

Before aggregating the reaction times for unjust, just, and neutral probes, we omitted the error trials in which participants did not complete the fragment correctly. Error rates were low for the different types of probes (4.49% for unjust probes, 5.77% for just probes, and 5.77% for neutral probes). In addition, we corrected for outliers by omitting individual reaction times that were faster than 500 ms or slower than 15,000 ms (11.86% for unjust probes, 11.86% for just probes, and 6.41% for neutral probes).

To test the effectiveness of our training procedure in inducing an interpretational readiness, we calculated a 2 (training procedure: training/control) × 3 (probe type: unjust/just/neutral) ANOVA with repeated measures on the second factor. We found a significant main effect of probe type, *F*_(2, 73)_ = 7.17, *p* < 0.01, η^2^ = 0.16, as well as a significant Probe × Training interaction effect, *F*_(2, 73)_ = 5.02, *p* < 0.01, η^2^ = 0.12 (see Table 1). Decomposing this interaction effect, there was a tendency that participants in the training condition reacted faster to unjust probes (*M* = 5716.47, *SD* = 1212.77) than participants in the control group (*M* = 6130.74, *SD* = 1196.27). However, this contrast was not significant, *t*_(75)_ = −1.51, *p* = 0.14, *d* = −0.35. In addition, there was a tendency that participants in the training condition reacted slower to neutral probes (*M* = 5640.35, *SD* = 1355.55) than participants in the control condition (*M* = 5168.86, *SD* = 1049.43), *t*_(75)_ = 1.69, *p* = 0.09, *d* = 0.39. For reaction times to just probes, there was no difference between the training condition (*M* = 5596.82, *SD* = 1107.32) and the control condition (*M* = 5683.54, *SD* = 1157.97), *t*_(75)_ = −0.34, *p* = 0.78, *d* = −0.01. Since the passages were not matched in length across probes types, we refrained from a comparison between probe types within one condition.

**Table 1:** **means and (standard deviations) of reactions times for unjust, just, and neutral probes, separately for the unjust training group and control group**.

**Reaction times**	**Unjust training group *M* (*SD*)**	**Control group *M* (*SD*)**
Unjust probes	5716 (1213)	6131 (1196)
Just probes	5598 (1107)	5684 (1158)
Neutral probes	5640 (1356)	5169 (1049)

Unjust probes = mean reaction times for unjust probe fragments (ms); just probes = mean reaction times for just probe fragments (ms); neutral probes = mean reaction times for neutral probe fragments (ms).

In order to investigate the specific effect of the training procedure regarding unjust interpretations over and above general effects on reaction times, we corrected for reaction times to neutral probes as a baseline when analyzing reaction times for unjust probes. We calculated difference terms by subtracting reaction times for neutral probes from reaction times for unjust probes and just probes, respectively. This way, it is possible to test for a specific effect of the unjust training condition on the readiness to resolve an ambiguity to imply an injustice over and above the general readiness to complete fragments.

We conducted a univariate ANOVAs with the factor training (training/control) and difference scores as dependent variables. In accordance with Hypothesis 1, we found significant main effects of the training on the difference score for unjust minus neutral probes, *F*_(1, 75)_ = 10.09, *p* < 0.01, η^2^ = 0.12, and on the difference score for just minus neutral probes, *F*_(1, 75)_ = 3.59, *p* = 0.051, η^2^ = 0.051. When corrected for reaction times for neutral probes as a baseline, participants in the training condition reacted significantly faster to unjust probes (*M* = 76.12, *SD* = 178.16) and to just probes (*M* = −43.53, *SD* = 176.75) than participants in the control condition (unjust probes: *M* = 876.44, *SD* = 178.16; just probes: *M* = 453.37, *SD* = 176.75).

### Effects of training on emotions

We tested for potential effects of the training procedure on emotional reactions toward the unequal allocation of money. Separate *t*-tests using condition as independent variable and outrage and guilt as dependent variables, did not yield any significant results for guilt, *t*_(75)_ = 0.61, *p* = 0.55, *d* = 0.13, or for outrage, *t*_(75)_ = −0.35, *p* = 0.73, *d* = −0.08.

### Altruistic compensation and punishment

We computed separate *t*-tests to investigate whether our training procedure affected behaviors in the mixed-game. For altruistic compensation, there was, consistent with Hypothesis 2a, a significant training effect, *t*_(75)_ = −2.34, *p* = 0.02, *d* = −0.55. Participants in the training condition invested on average approximately 1 € more (*M* = 5.29, *SD* = 1.99) to compensate Person B than participants in the control condition (*M* = 4.33, *SD* = 1.56). In accordance with Hypothesis 2b, there was no significant effect of the training on altruistic punishment, *t*_(75)_ = 0.47, *p* = 0.64, *d* = −0.11. Participants in the unjust training condition invested a similar amount of money to reduce the outcome of Person A (*M* = 5.89, *SD* = 1.74) compared to those in the control condition (*M* = 6.10, *SD* = 2.10).

## Discussion

The goal of our study was to investigate the psychological processes that underlie altruistic compensation by identifying a specific interpretational pattern and testing its role in causing this behavioral reaction. We found evidence that interpretational processes in ambiguous social situations are relevant for explaining why some people invest their own resources to compensate a victim of another person's norm violations.

We successfully adapted a training procedure from anxiety research that was effective at experimentally inducing a tendency to interpret one's own better outcome as unjustified (Hypothesis 1). Moreover, our results showed that the training procedure systematically increased altruistic compensation (Hypothesis 2a). Compared with the control condition, participants trained to interpret their own better outcome as unjustified invested more of their own resources to compensate another person who was disadvantaged by a third person. Importantly, the behavioral effect of the training cannot be attributed to emotional effects of the procedure. Our study complements prior research on downward social comparison in important ways. Whereas downward comparison can decrease prosocial behaviors (Yip and Kelly, 2013), our findings suggest that this process can also serve to enhance altruistic compensation, depending on whether one interprets one's own better outcome as unjustified. As expected (Hypothesis 2b), there was no effect of the training on altruistic punishment. For punishment to occur, a focus on the perpetrator is necessary (Darley and Pittman, 2003). By contrast, altruistic compensation depends on the interpretation of one's standing relative to the victim.

For future research, the investigation of chronic interpretational patterns underlying personality dispositions could shed further light on the relevance of these processes for prosocial behavior. As explained above, justice sensitivity (Schmitt et al., 2010) has been found to be correlated with interpretational biases (Baumert and Schmitt, 2009; Baumert et al., 2012) and to predict altruistic compensation (Lotz et al., 2011) as well as other forms of prosocial behavior (e.g., Gollwitzer et al., 2005; Baumert et al., 2013). Accordingly, it seems possible that this disposition modulates the individual effectiveness of the training procedure for changing interpretational patterns and enhancing altruistic compensation. Specifically, justice sensitivity has been differentiated according to the perspectives from which injustice can be experienced: as a victim, as an observer, as beneficiary or as perpetrator (Mikula, 1994). One could assume that high victim sensitives are less susceptible for training effects than low victim sensitives, because they tend to interpret own advantages as compensation for past injustice. By contrast, high beneficiary sensitives could respond stronger to the training procedure than low beneficiary sensitives because these people tend to see an own better outcome as unjustified, a pattern that corresponds to the content of the training sentences.

### Limitations

To improve future studies, we will note several limitations of the present research.

First, the measurement of the interpretational tendency could be improved. Unjust, just, and neutral probes were not perfectly matched in length. Therefore, the comparison of reaction times across the three probe types was difficult to interpret. It is possible that the main effect of probe type, with unjust probes eliciting longer reaction times than just and neutral probes, resulted from the unequal length of the passages rather than from the specific content. Matching the probes more closely by length will allow for more specific conclusions.

More importantly, just probes were sometimes rated as neutral on the pretest despite further modification of these passages. Thus, the just content should be made more salient to obtain greater differences in the ratings between just and neutral probes. However, it seems possible that just situations are generally experienced as normal and therefore not perceived as distinct from neutral passages. By contrast, our pretests suggest that the unjust passages are perceived as clearly unjust and distinct from neutral situations, despite the non-significant interrater agreement.

Second, further strategies for the induction of interpretational tendencies could be investigated. In our study, we employed a training procedure based on the assumption that the active completion of fragments, which leads to an unjust connotation of the ambiguous passage, induces a readiness to interpret one's own advantages as unjustified. However, the possibility that a passive priming without fragment completion might have a similar effect cannot be excluded. In other words, merely reading the training passages might also induce an interpretational tendency. In/justice-related concepts might be activated during reading, guiding the interpretation of ambiguous situations (e.g., Erdley and D'Agostino, 1988) in such a way that one's own advantage is seen as unjustified. Introducing a priming condition without fragment completion in future studies will allow for the investigation of this option. Importantly, if this condition has the same effects on reaction times and behavior in the mixed-game as our training procedure, this finding would further confirm the causality of the proposed interpretational tendency for altruistic compensation. Furthermore, the inclusion of a just training condition, where ambiguous sentences have to be resolved to imply justice could allow more specific conclusions regarding behavioral reactions. Possibly, the induced tendency to interpret own advantages as justified could attenuate prosocial behavior.

Third, future research could investigate the permanence of our training's effect on altruistic compensation. For practical purposes, knowledge about the stability of interventions that enhance this kind of prosocial behavior is necessary. In a longitudinal study, the training and priming procedures described above could be compared on the stability of their effects. The effect of our training procedure that included an active completion of the fragments on altruistic compensation might last longer than the effect elicited via a passive priming procedure.

Fourth, it would be interesting for further studies to investigate gender differences in response to the training procedure. In our sample, such a comparison is not possible due to the unequal proportion of males and females. Future studies should overcome this limitation.

### Conclusion

Despite its limitations, the present research is an important step toward understanding the psychological processes that cause altruistic compensation. We were able to experimentally manipulate interpretational patterns with regard to injustice in ambiguous social situations. The tendency to interpret one's own advantages as unjustified was induced by a training procedure and in turn enhanced the amount of one's own resources that a person invested to compensate a victim of a norm violation. By improving and further testing this training procedure, appropriate interventions can be designed to enhance prosocial behavior—in particular, altruistic compensation in bystander situations that require an intervention in which people must invest their own resources in order to restore justice.

### Conflict of interest statement

This research was supported by a grant from the German Research Foundation, No. SCHM 1092-10/3.
